# Supplementary material for: Advanced Glycation End-Products (AGEs) of Lysine and Effects of Anti-TCR/Anti-TNF-α Antibody-Based Therapy in the LEW.1AR1-iddm Rat, an Animal Model of Human Type 1 Diabetes
Source: Int J Mol Sci. 2022 Jan 28;23(3):1541. doi: 10.3390/ijms23031541 (PMC8915180; doi:10.3390/ijms23031541)
Supplement: Supplementary file 1 [file ijms-23-01541-s001.zip › ijms-1503308 - supplementary.pdf]

Multivariate statistical analysis

Multivariate statistical analysis was performed in all study samples analysed, i.e., for the tissue weight-corrected content of free and proteinic amino acids and for the molar ratio of proteinic-to-free amino acids. The results of these analyses are summarized in the following Tables and Figures and discussed below.

A) Results

Table S1. Overview of the PCA results for the study groups (analytes: glucose, CML, furosine, lysine, tyrosine).

			**(A) Pancreas**		
	**Component**	**Eigenvalue**	**Difference**	Proportion	Cumulative
**ngCO**	PC1	2.54469	0.855678	0.5089	0.5089
	PC2	1.68902	1.25533	0.3378	0.8467
**acTID**	PC1	2.75317	0.869431	0.5506	0.5506
	PC2	1.88374	1.60297	0.3767	0.9274
**chTID**	PC1	3.68286	2.56156	0.7366	0.7366
	PC2	1.1213	0.925471	0.2243	0.9608
**cuTID**	PC1	3.85037	2.81491	0.7701	0.7701
	PC2	1.03546	0.921282	0.2071	0.9772
			**(B) Kidney**		
	Component	Eigenvalue	Difference	Proportion	Cumulative
**ngCO**	PC1	3.14701	1.6769	0.6294	0.6294
	PC2	1.4701	1.16971	0.2940	0.9234
**acTID**	PC1	3.20808	1.94758	0.6416	0.6416
	PC2	1.2605	0.762124	0.2521	0.8937
**chTID**	PC1	4.28005	3.70162	0.8560	0.8560
	PC2	0.578423	0.436891	0.1157	0.9717
**cuTID**	PC1	2.73619	0.899182	0.5472	0.5472
	PC2	1.837	1.41019	0.3674	0.9146
			**(C) Spleen**		
	Component	Eigenvalue	Difference	Proportion	Cumulative
**ngCO**	PC1	2.84246	1.88646	0.5685	0.5685
	PC2	0.955999	0.292721	0.1912	0.7597
**acTID**	PC1	2.74181	1.08814	0.5484	0.5484
	PC2	1.65367	1.17761	0.3307	0.8791
**chTID**	PC1	2.72023	1.18304	0.5440	0.5440
	PC2	1.53719	0.794613	0.3074	0.8515
**cuTID**	PC1	3.51976	2.14543	0.7040	0.7040
	PC2	1.37433	1.26842	0.2749	0.9788

Table S2. Statistically significant free amino acids highlighted after pairwise comparison of the examined groups in all organ tissues, along with the respective variable importance for the projection (VIP) values, q-values (FDR correction) and the logarithm base two of the fold change log2FC.

	5-OH-K_L	ADMA	Ala	Arg	Asp+Asn	CML	Glu/Gln	Gly	Leu/Ile	Lys	Met		OH-Pro	Orn/Cit	Phe	Pro	Ser	Thr	Trp	Tyr	Val
acTID *vs* chTID	**PANCREAS** VIP	-	-	-	-	-	1.25	1.2	1.11	1.32	1.2	1.28	1.41	-	-	1.01	-	-	-	-	-
	qvalue	-	-	-	-	-	4.7E-02	3.3E-02	3.0E-02	4.0E-03	1.6E-02	7.0E-03	7.0E-05	-	-	5.0E-02	-	-	-	-	-
	log2FC	-	-	-	-	-	1.46	0.74	0.73	0.38	0.69	1.09	1.61	-	-	0.6	-	-	-	-	-
acTID *vs* cuTID	VIP	-	-	-	-	-	1.25	1.33	1.21	1.4	1.34	1.26	1.29	-	-	1.2	-	-	-	-	1.3
	qvalue	-	-	-	-	-	3.8E-02	3.0E-03	8.0E-03	1.0E-04	1.0E-03	6.0E-03	2.0E-03	-	-	8.0E-03	-	-	-	-	2.0E-03
	log2FC	-	-	-	-	-	1.61	1.25	0.5	0.64	1.36	1.04	0.97	-	-	0.84	-	-	-	-	1.12
acTID *vs* chTID	**KIDNEY** VIP	1.3	-	1.32	-	1.25	-	-	1.16	1.28	1.22	-	-	1.25	1.28	-	1.17	1.22	-	1.25	1.27
	qvalue	2.0E-04	-	3.0E-05	-	3.0E-04	-	-	9.4E-03	2.3E-04	1.5E-03	-	-	4.7E-04	1.0E-04	-	3.4E-03	8.3E-04	-	2.9E-04	1.3E-04
	log2FC	1.6	-	1.1	-	0.98	-	-	0.4	1.1	1.37	-	-	1.49	0.79	-	0.65	1.17	-	0.7	0.38
acTID *vs* cuTID	VIP	1.15	-	1.32	-	1.21	-	-	-	1.27	1.22	-	-	1.22	1.24	-	-	1.21	-	-	1.26
	qvalue	4.5E-02	-	7.0E-04	-	1.2E-02	-	-	-	1.9E-03	5.3E-03	-	-	4.6E-03	2.2E-03	-	-	4.0E-03	-	-	1.0E-03
	log2FC	0.66	-	1.06	-	0.72	-	-	-	1.0	1.22	-	-	1.36	0.68	-	-	1.04	-	-	0.2
acTID *vs* chTID	**SPLEEN** VIP	-	1.27	-	1.29	1.21	-	1.26	-	1.13	1.16	1.28	-	1.18	1.13	1.29	1.21	1.27	-	1.25	1.19
	qvalue	-	6.3E-04	-	4.7E-05	2.2E-03	-	2.6E-04	-	9.3E-03	3.9E-03	2.8E-05	-	2.0E-03	5.0E-03	8.9E-06	6.0E-04	4.4E-05	-	1.1E-04	8.4E-04
	log2FC	-	1.31	-	0.77	0.6	-	0.76	-	0.3	0.39	0.64	-	0.39	0.31	0.6	0.34	0.73	-	0.5	0.48
acTID *vs* cuTID	VIP	-	1.35	-	1.33	-	-	-	-	-	1.3	1.31	-	-	-	1.26	-	1.33	1.23	-	1.29
	qvalue	-	1.9E-03	-	1.3E-03	-	-	-	-	-	1.7E-03	1.2E-03	-	-	-	3.4E-03	-	5.0E-04	4.9E-03	-	1.3E-03
	log2FC	-	1.11	-	0.64	-	-	-	-	-	0.42	0.34	-	-	-	0.42	-			-	0.45

Table S3. Statistically significant proteinic amino acids highlighted after pairwise comparison of the examined groups in all organ tissues, along with the respective variable importance for the projection (VIP) values, q-values (FDR correction) and the logarithm base two of the fold change log2FC.

		5-OH-K_D	5-OH-K_L	ADMA	Ala	Arg	Asp+Asn	CML	Fu-rosin	Glu+Gln	Gly	Leu+Ile	Lys	Met	MMK	OH-Pro	Orn+Cit	Phe	Pro	Sar-cosin	Ser	Thr	Trp	Val
	**PAN-CREAS**																							
**ngCo vs acTID**	**VIP**	1.32	1.36	1.08	1.03	-	-	-	-	-	1.16	1.19	1.28	-	-	1.22	-	1.05	1.10	-	1.32	1.25	-	1.08
	**qvalue**	6.3E-03	7.9E-03	3.3E-02	3.8E-02	-	-	-	-	-	1.9E-02	1.5E-02	8.2E-03	-	-	1.3E-02	-	3.4E-02	3.0E-02	-	8.9E-03	1.0E-02	-	3.0E-02
	**log2FC**	0.89	0.99	-0.45	-0.23	-	-	-	-	-	-0.24	-0.25	-0.26	-	-	0.73	-	-0.25	-0.22	-	-0.38	-0.26	-	-0.23
**ngCo vs chTID**	**VIP**	-	-	-	-	-	-	-	-	-	-	-	-	-	-	1	-	-	-	-	-	-	1.95	-
	**qvalue**	-	-	-	-	-	-	-	-	-	-	-	-	-	-	0.05	-	-	-	-	-	-	0.03	-
	**log2FC**	-	-	-	-	-	-	-	-	-	-	-	-	-	-	-1.50	-	-	-	-	-	-	0.59	-
	**KID-NEY**																							
**acTID vs chTID**	**VIP**	1.56	1.52	1.52	-	-	-	-	-	-	-	-	1.28	-	-	1.35	-	-	-	-	-	-	1.41	-
	**qvalue**	3.3E-05	3.2E-04	2.4E-04	-	-	-	-	-	-	-	-	2.6E-02	-	-	1.3E-05	-	-	-	-	-	-	4.3E-03	-
	**log2FC**	-1.93	-1.76	0.94	-	-	-	-	-	-	-	-	0.35	-	-	-2.23	-	-	-	-	-	-	0.83	-
**acTID vs cuTID**	**VIP**	1.09	-	1.17	1.04	1.08	1.18	-	-	1.09	1.06	1.13	1.26	-	-	1.25	1.08	1.14	1.07	-	1.16	1.12	-	1.18
	**qvalue**	6.0E-03	-	1.9E-03	9.2E-03	6.1E-03	2.3E-03	-	-	6.0E-03	7.2E-03	3.6E-03	1.6E-04	-	-	2.1E-04	6.4E-03	3.5E-03	6.7E-03	-	2.2E-03	4.3E-03	-	2.2E-03
	**log2FC**	0.51	-	-0.87	-0.36	-0.59	-0.65	-	-	-0.55	-0.42	-0.47	-0.56	-	-	1.85	-0.60	-0.47	-0.43	-	-0.43	-	-	-0.57
	**SPLEEN**																							
**ngCo vs chTID**	**VIP**	-	1.47	1.54	-	-	-	-	1.54	-	-	-	-	1.28	-	-	-	-	-	1.43	1.40	-	1.43	-
	**qvalue**	-	6.3E-03	3.4E-03	-	-	-	-	1.8E-03	-	-	-	-	3.3E-02	-	-	-	-	-	8.5E-03	8.8E-03	-	7.6E-03	-
	**log2FC**	-	-0.53	-0.70	-	-	-	-	1.19	-	-	-	-	-0.4	-	-	-	-	-	-1.47	-0.32	-	-0.84	-
**acTID vs chTID**	**VIP**	1.34	1.49	1.55	-	-	-	1.30	1.53	-	-	-	-	1.52	1.36	-	-	-	-	1.43	-	-	1.54	-
	**qvalue**	2.0E-02	3.8E-03	5.1E-03	-	-	-	2.4E-02	3.1E-03	-	-	-	-	3.0E-03	1.7E-02	-	-	-	-	8.3E-03	-	-	3.5E-03	-
	**log2FC**	-0.54	-0.57	-0.65	-	-	-	0.74	1.42	-	-	-	-	-0.2	0.64	-	-	-	-	-1.33	-	-	-0.79	-

Table S4. Statistically significant molar ratio of proteinic to free amino acids found after pairwise comparison of the examined groups in all organ tissues, along with the respective variable importance for the projection (VIP) values, q-values (FDR correction) and the logarithm base two of the fold change log2FC.

		5-OH-K_D	5-OH-K_L	ADMA	Ala	Arg	Asp+Asn	Fu-rosin	GAA	Glu+Gln	Gly	Leu+Ile	Lys	Met	OH-Pro	Orn+Cit	Phe	Pro	Sar-cosin	Ser	Thr	Tyr	Val
ngCo vs chTID	PAN-CREAS VIP	1.59	1.77	-	-	-	-	-	-	-	-	-	-	-	1.77	-	-	-	1.95	-	-	-	-
	qvalue	4.8E-02	1.5E-02	-	-	-	-	-	-	-	-	-	-	-	1.0E-02	-	-	-	5.1E-04	-	-	-	-
	log2FC	0.70	0.66	-	-	-	-	-	-	-	-	-	-	-	0.40	-	-	-	1.51	-	-	-	-
acTID vs chTID	VIP	-	-	-	-	-	-	-	-	-	-	-	-	-	-	-	-	-	1.98	-	-	-	-
	qvalue	-	-	-	-	-	-	-	-	-	-	-	-	-	-	-	-		2.4E-03	-	-	-	-
	log2FC	-	-	-	-	-	-	-	-	-	-	-	-	-	-	-	-	-	1.22	-	-	-	-
acTID vs cuTID	VIP	-	-	-	-	-	-	-	1.95	-	-	-	-	-	-	1.39	-	-	1.99	-	-	-	-
	qvalue	-	-	-	-	-	-	-	3.0E-03	-	-	-	-	-	-	5.0E-02	-	-	9.8E-03	-	-	-	-
	log2FC	-	-	-	-	-	-	-	-0.74	-	-	-	-	-	-	-0.58	-	-	-0.97	-	-	-	-
ngCo vs acTID	KID-NEY VIP	1.71	-	-	-	-	-	1.63	-	-	-	-	-	-	-	1.33	-	-	-	1.39	-	-	-
	qvalue	1.8E-04	-	-	-	-	-	1.2E-03	-	-	-	-	-	-	-	4.5E-02	-	-	-	3.3E-02	-	-	-
	log2FC	0.93	-	-	-	-	-	0.89	-	-	-	-	-	-	-	-0.15	-	-	-	-0.36	-	-	-
ngCo vs chTID	VIP	-	-	-	1.32	1.08	1.30	1.21	-	-	-	1.26	1.4	-	1.22	1.39	1.08	-	-	-	1.21265	-	1.28
	qvalue	-	-	-	4.5E-03	3.9E-02	5.0E-03	1.2E-02	-	-	-	7.4E-03	8.9E-04	-	1.2E-02	6.8E-04	4.1E-02	-	-	-	1.2E-02	-	6.0E-03
	log2FC	-	-	-	0.78	0.67	0.75	0.73	-	-	-	0.74	1.05	-	-0.54	0.64	0.53	-	-	-	0.74	-	1.01

acTID vs cuTID	VIP	1.06	-	-	1.18	1.20	1.20	1.17	-	1.11	1.14	1.21	1.19	1.04	1.13	1.12	1.21	1.09	-	1.15	1.21	1.15	1.18
	qvalue	3.5E-03	-	-	2.3E-04	1.2E-04	1.4E-04	3.4E-04	-	1.6E-03	7.7E-04	2.7E-04	2.1E-04	5.0E-03	9.7E-04	1.1E-03	1.7E-04	2.2E-03	-	6.1E-04	1.6E-04	6.1E-04	2.3E-04
	log2FC	0.54	-	-	-1.24	-1.26	-1.28	0.71	-	-0.68	-0.47	-1.39	-1.67	-0.33	0.46	-0.91	-1.07	-0.74	-	-0.89	-1.38	-1.0	-1.75
acTID vs chTID	VIP	1.28	-	-	1.24	1.17	1.27	-	-	-	-	1.27	1.29	-	1.24	1.28	1.23	-	-	1.15	1.21	-	1.26
	qvalue	8.6E-05	-	-	2.8E-04	1.8E-03	1.1E-04	-	-	-	-	9.6E-05	6.8E-05	-	2.8E-04	1.3E-04	4.1E-04	-	-	2.5E-03	5.7E-04	-	1.3E-04
	log2FC	-	-	-	0.32	0.31	0.36	-	-	-	-	0.37	0.47	-	-0.22	-	-	-	-	0.22	0.37	-	0.46
	SPLEEN																						
ngCo vs chTID	VIP	-	-	-	-	1.39	1.37	-	1.33	1.29	-	-	1.28	1.15	1.14	-	-	1.22	-	-	1.22	-	1.18
	qvalue	-	-	-	-	2.0E-04	4.9E-04	-	1.3E-03	3.1E-03	-	-	2.9E-03	1.6E-02	1.6E-02	-	-	9.0E-03	-	-	8.0E-03	-	1.2E-02
	log2FC	-	-	-	-	0.52	0.89	-	-0.58	0.40	-	-	0.52	0.2	-0.31	-	-	0.4	-	-	0.49	-	0.59
acTID vs chTID	VIP	-	-	1.05	-	1.31	1.26	-	1.26	1.21	1.03	-	1.16	1.22	1.12	1.12	-	1.18	-	-	1.20	-	1.13
	qvalue	-	-	1.5E-02	-	2.1E-05	4.6E-04	-	4.9E-04	1.6E-03	1.8E-02	-	3.2E-03	1.4E-03	5.9E-03	5.7E-03	-	2.7E-03	-	-	1.7E-03	-	5.5E-03
	**log2FC**	**-**	**-**	**0.6**	**-**	**0.67**	**0.67**	**-**	**-0.5**	**0.54**	**0.17**	**-**	**0.45**	**0.33**	**-0.39**	**0.37**	**-**	**0.44**	**-**	**-**	**0.64**	**-**	**0.55**
**acTID vs cuTID**	**VIP**	**-**	**-**	**1.19**	**1.04**	**1.32**	**1.09**	**-**	**1.28**	**1.01**	**1.00**	**1.22**	**1.27**	**-**	**1.23**	**-**	**1.01**	**1.15**	**-**	**-**	**1.20**	**-**	**1.25**
	**qvalue**	**-**	**-**	**2.9E-03**	**2.3E-02**	**1.2E-04**	**1.2E-02**	**-**	**6.3E-04**	**2.7E-02**	**2.5E-02**	**1.8E-03**	**5.9E-04**	**-**	**1.4E-03**	**-**	**2.6E-02**	**5.4E-03**			**2.4E-03**	**-**	**8.6E-04**
	**log2FC**	**-**	**-**	**-0.87**	**-0.26**	**-0.73**	**-0.42**	**-**	**0.71**	**-0.36**	**-0.2**	**-0.34**	**-0.55**	**-**	**0.6**	**-**	**-0.23**	**-0.41**	**-**	**-**	**-0.57**	**-**	**-0.6**

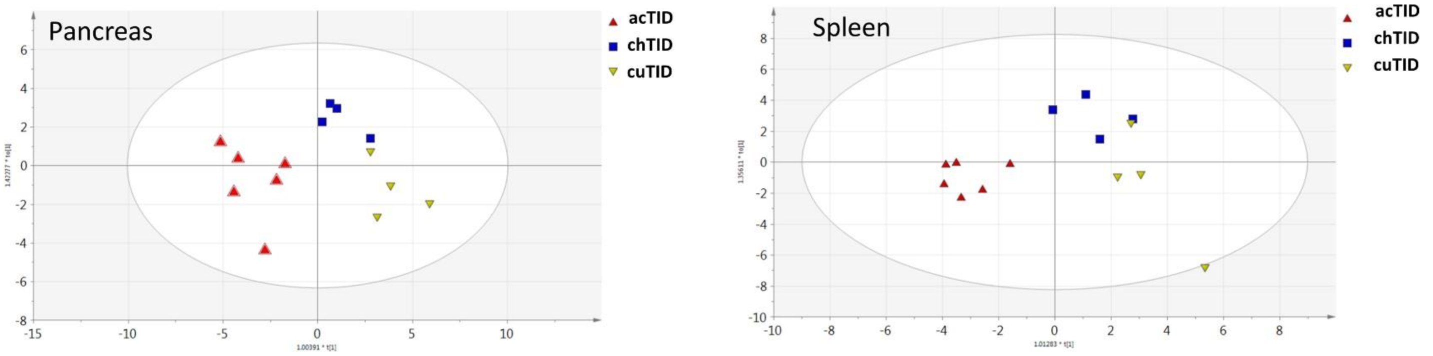

Figure S1. Left panel: OPLS score plot of acTID, chTID and cuTID groups in pancreas for free amino acids. Right panel: OPLS score plot of acTID, chTID and cuTID groups in spleen for free amino acids.

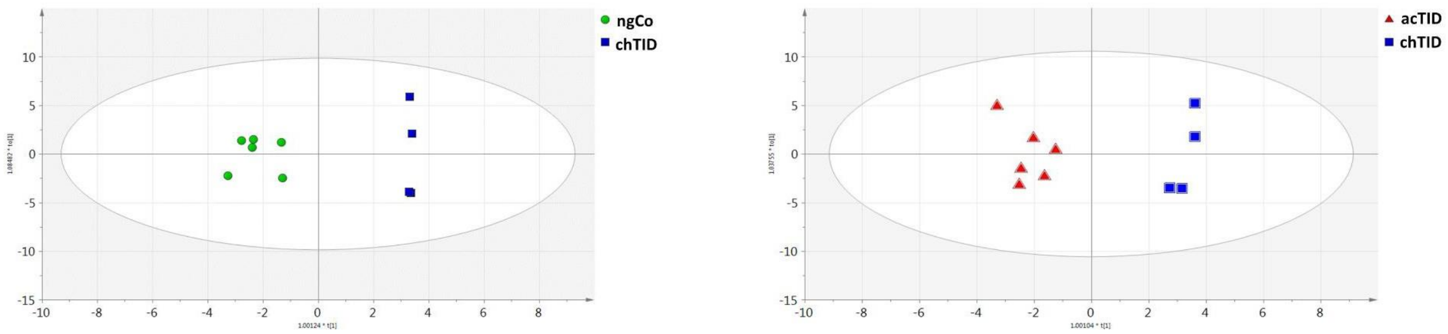

Figure S2. OPLS-DA score plots of ngCo vs chTID (left panel) and acTID vs chTID (right panel) in spleen for proteinic amino acids.

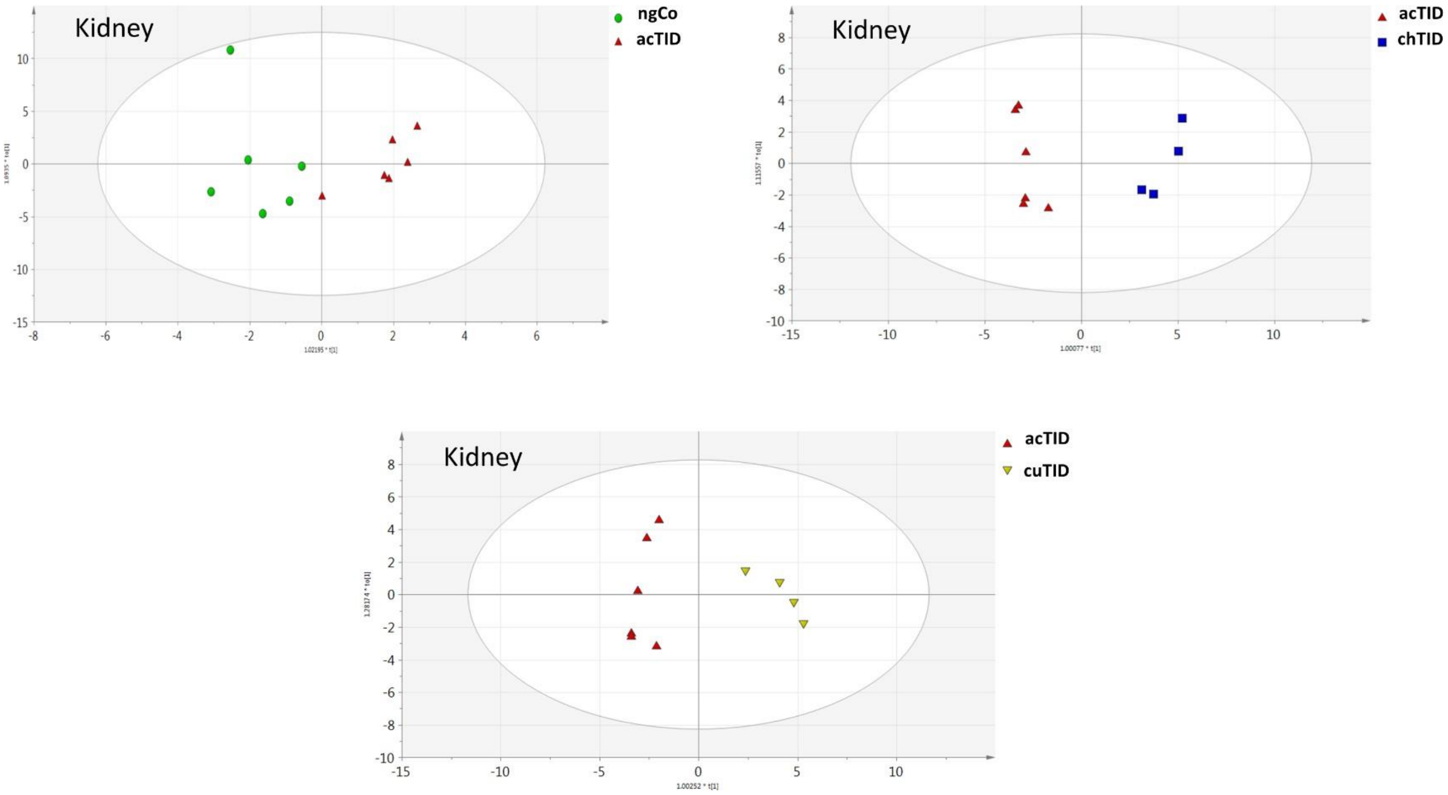

Figure S3. OPLS-DA score plots of ngCo vs acTID, acTID vs chTID, and acTID vs cuTID groups in kidney for free amino acids.

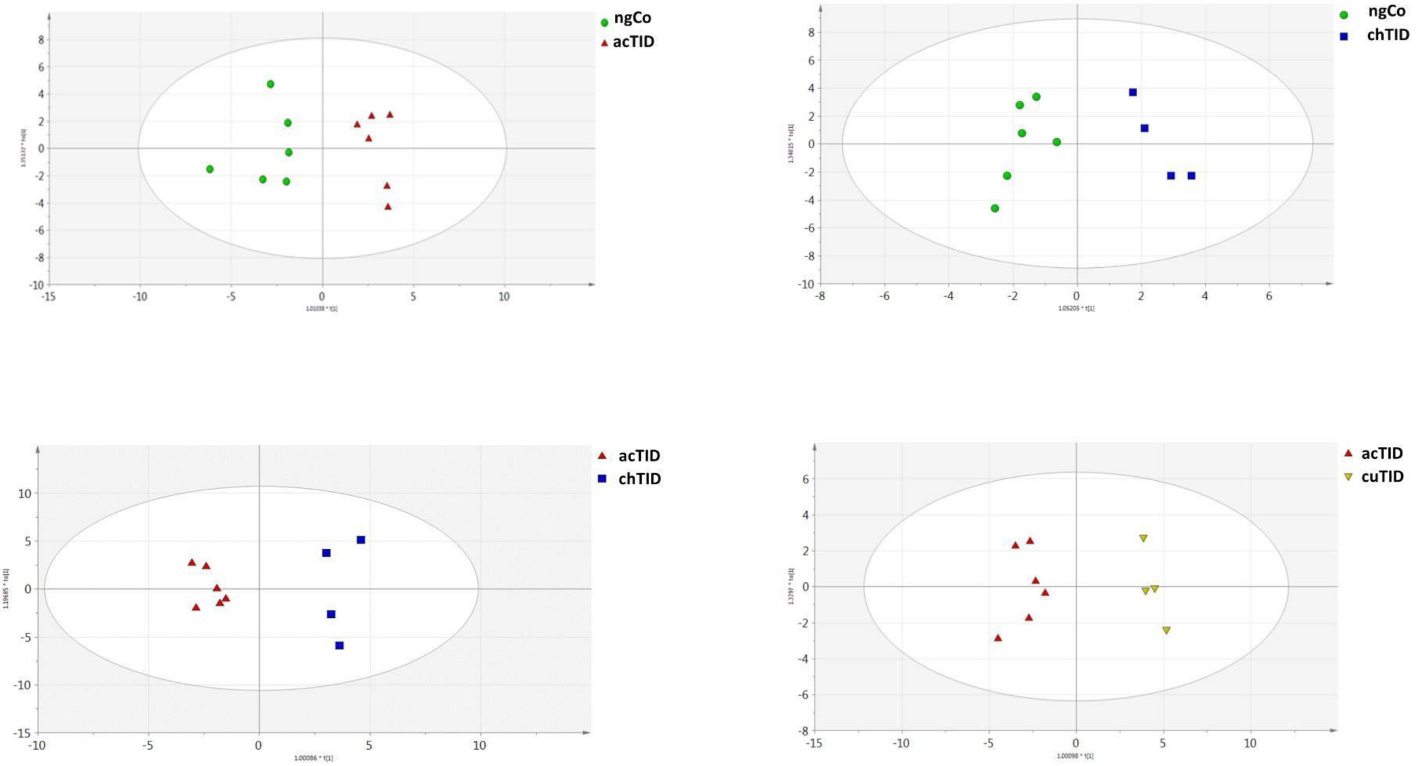

Figure S4. OPLS-DA score plots of ngCo vs acTID, ngCo vs chTID, acTID vs chTID, and acTID vs cuTID groups in kidney for proteinic amino acids.

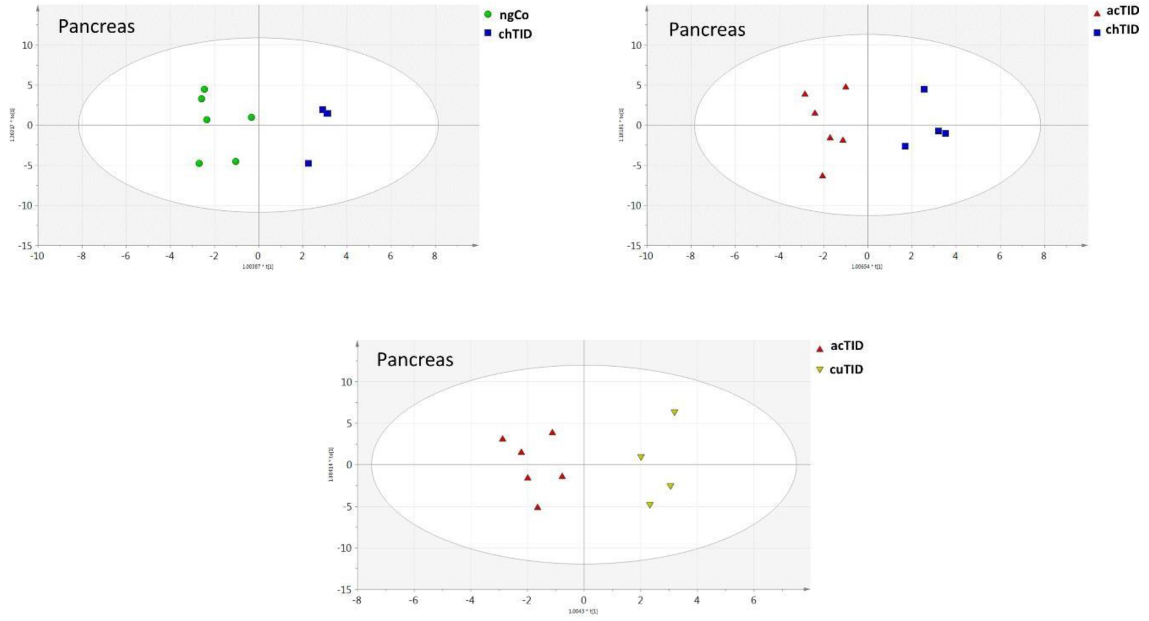

Figure S5. OPLS-DA score plots of the ngCo vs chTID, acTID vs chTID and acTID vs cuTID groups in pancreas for the molar ratio.

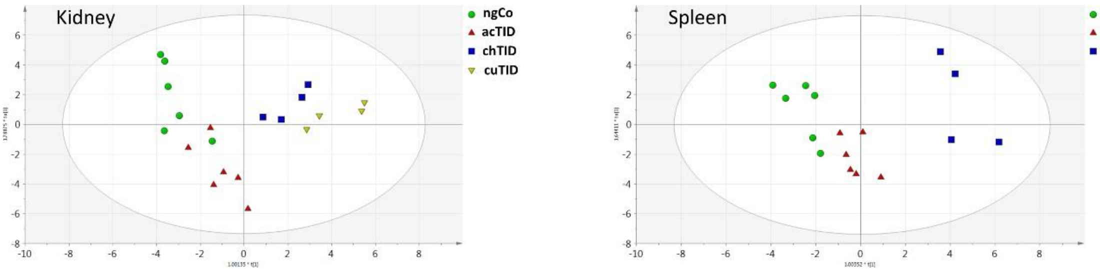

Figure S6. Left panel: OPLS score plot of all examined groups in kidney for the molar ratio. Right panel. OPLS score plot of the ngCo, acTID and chTID groups in spleen for the molar ratio.

Discussion

Regarding free amino acids of the pancreas, orthogonal partial least squares discriminant analysis (OPLS-DA) showed a clear discrimination between theacTID group and chTID and cuTID groups. Both score plots models illustrated satisfactory R2X, Q2Y and R2Y values, while both CV-ANOVAs being < 0.05 (Table S2). Figure S1 (left) illustrates an OPLS model constructed with the respective groups. Loadings plots and variable importance for the projection (VIP) plots highlighted Leu/Ile, OH-Pro, Met, Glu/Gln, Lys and Val as the most affected metabolites among groups (VIP >1.3). In contrast, proteinic amino acids did not show any statistically significant differentiation among the examined groups.

In the spleen, free amino acids were only significantly altered between the acTID group and chTID and cuTID groups. Both constructed OPLS-DA models, illustrated a statistically strong differentiation between the pairwise compared groups, with CV-ANOVA p-values being <0.02. In both cases, more than 10 amino acids and derivatives presented altered, such as ADMA, Pro, Arg, Met and Thr with all having VIP values >1.3 (Table S2). Figure S1 (right) illustrates an OPLS score plot model of the three examined groups (R2X 0.736, R2Y 0.853, Q2Y 0.646). Looking into the data of the proteinic amino acid content in spleen, significant differences were observed between ngCo and chTID (R2X 0.788, R2Y 0.96, Q2Y 0.92), as well as between the acTID and chTID (R2X 0.842, R2Y 0.96,Q2Y 0.917) group (Fig. S2). Among important compounds were ADMA, furosine, 5-OH-K_L, sarcosine and Trp (Table S3).

In the kidney, free amino acids were found to be strongly differentiated again between the acTID and chTID, acTID and cuTID groups (Fig. S3). Cross- validation parameters were found in acceptable values (not overfitting). Ala, 5-OH-K_L, Phe and Asp+Asn were significantly higher in acTID group. On the other hand, proteinic amino acids showed more statistically significant variations between the groups. More specifically, constructed multi-variate models presented statistical differentiation between ngCo vs acTIC (R2X 0.775, R2Y 0.868, Q2Y 0.683), ngCo vs chTID (R2X 0.559, R2Y 0.91, Q2Y 0.84), acTID vs chTID (R2X 0.872, R2Y 0.96, Q2Y 0.93) and acTID vs cuTID (R2X 0.783, R2Y 0.958, Q2Y 0.813) groups (Fig. S4). Table S4 presents the altered proteinic amino acids in the groups.

In the liver, no differences were observed for free or proteinic amino acids (data not shown).

The molar ratio of proteinic-to-free amino acids in the study groups was statistically accessed in all organ examined. In the pancreas, multivariate statistical analysis and in extend OPLS-DA score plot models (Fig. S5) illustrated a strong differentiation between acTID vs cuTID groups (CV-ANOVA, 0.03) and a mild one when comparing ngCo with chTID (CV-ANOVA, 0.06), and acTID with chTID (CV-ANOVA, 0.05). 5-OH-K, OH-Pro, sarcosine and Orn/Cit were found to be statistically differentiated among groups (Table S4).

In the kidney, the statistical sigignifance of the molar ratio of proteinic-to-free amino acids was evident by PCA and OPLS (Fig. S6) of all studied groups,except when comparing chTID vs cuTID group. Models' characteristics validated the goodness of fit in both X and Y variables, as all presented R2Y(cum) andQ2 values >0.851 and >0.765, respectively,

while p-values of Cross Validated ANOVA Analysis were < 0.026. According to VIP, discrimination between the groups was due to Ala, orn+cit, OH-pro, 5-OH-K, Lys, furosine, Phe, Ser and Val, markers that presented VIP values >1 (Table S4).

Using the data collected from spleen, multivariate statistical analysis illustrated significant differentiations between the examined groups. More specifically, OPLS managed to separate ngCo vs chTID, as well as acTID vs both chTID and cuTID groups (Fig. S6). In total, sixteen amino acids were found to differ (Table S4).
